# Implementation of a cardiovascular toolkit in primary care increased women Veterans’ engagement in behavior change programs: results from a non-randomized stepped wedge trial

**DOI:** 10.21203/rs.3.rs-5347756/v1

**Published:** 2024-11-26

**Authors:** Melissa M Farmer, Alison B Hamilton, Erin P Finley, Martin Lee, Catherine Chanfreau, Clarie Than, Julian Brunner, C. Amanda Schweizer, Alexis K Huynh, Tannaz Moin, Bevanne Bean-Mayberry

**Affiliations:** VA Greater Los Angeles Healthcare System; VA Greater Los Angeles Healthcare System; VA Greater Los Angeles Healthcare System; VA Greater Los Angeles Healthcare System; VA Salt Lake City Healthcare System; VACO; VA Greater Los Angeles Healthcare System; VA Greater Los Angeles Healthcare System; VA Greater Los Angeles Healthcare System; VA Greater Los Angeles Healthcare System; VA Greater Los Angeles Healthcare System

**Keywords:** Health Promotion/Prevention/Screening, Modeling: Multi-level, VA Health Care System, Primary Care, Gender/Sex Differences in Health and Health Care, veterans, women

## Abstract

**Background:**

Cardiovascular (CV) disease is the leading cause of death among U.S. women, yet women have a limited understanding of their CV-related morbidity and mortality risks. Provider-, system-, and patient-level barriers point to a need for multi-level evidence-based strategies to facilitate CV risk reduction. Guided by the Replicating Effective Programs framework and complexity theory, we implemented a CV Toolkit in primary care clinics for women Veterans. The objective was to evaluate the effect of implementation of CV toolkit on participation in behavior change programs that target CV risk reduction.

**Methods:**

In partnership with the Veterans Health Administration (VA) Office of Women’s Health and National Center for Health Promotion and Disease Prevention, we developed and implemented a CV Toolkit at five geographically diverse VA sites between March 2017-March 2020. Using a non-randomized stepped wedge design, we evaluated the effect of the implementation of toolkit on participation in the VA MOVE! weight management program, and on participation in health promotion and disease prevention (HPDP) programs (coaching, facilitated groups, etc.) and/or complementary integrative health (CIH) programs (yoga, meditation, etc.). We utilized a three-level (patient, site, time) non-linear fixed effect model with stratification by age (65 and older versus younger). Patient participation, utilization, and demographics were extracted from VA administrative data for all women with at least one primary care visit at a participating site from December 2016-March 2020 (n = 6009).

**Results:**

Women were on average 45 years old; 38% were white, 31% Black, 17% Hispanic; and over a third had CV risk factors and/or mental health diagnoses. For women 65 years and older (n = 540), active toolkit implementation resulted in increased odds of MOVE! participation (OR = 1.09; 95% CI:1.030–1.152) compared to when the toolkit was not active either within or between sites. Women younger than 65 (n = 5469) had increased odds of using HPDP/CIH programs during active toolkit implementation (OR = 1.01; 95% CI:1.002–1.022).

**Conclusions:**

Active implementation of the CV Toolkit intervention was significantly associated with increasing participation in behavior change programs. Variation in program participation by age suggests that the diversity of behavior change programs available to women Veterans may facilitate participation across the lifespan.

**Trial registration:**

Clinical Trials.gov, NCT02991534. Registered 12-09-2016, https://clinicaltrials.gov/study/NCT02991534?cond=NCT02991534&rank=1

## BACKGROUND

Cardiovascular (CV) disease is the leading cause of death among women in the United States, causing one in three deaths each year when combining heart disease and stroke,^1-3^ yet women have a limited understanding of their CV-related morbidity and mortality risks.^4^ With a national focus on improving CV awareness and knowledge, the American Heart Association (AHA) found that US women’s rates of CV awareness improved from 30% in 1997 to 54% in 2009 but then stagnated by 2012, with a minimal increase to only 56%.^4,5^ Even more disturbing are findings of a sharp 10-year decline in knowledge of heart disease as the leading cause of death for American women, from 65% aware in 2009 to 44% in 2019.^6^ In terms of CV risk factors, women suffer disparities in risk factor control (e.g., blood pressure, cholesterol, and intermediate diabetes outcomes),^7-11^ and have higher rates of obesity and physical inactivity than men,^12^ likely contributing to the increasing prevalence of coronary heart disease in women.^13-17^

While multiple CV guidelines are available to aid in controlling women’s CV risk factors, guideline-concordant practices are difficult to achieve in routine care due to provider-, system-, and patient-level barriers. Provider- and system-level barriers include lack of time during appointments, lack of awareness of and difficulties interpreting the latest CV prevention guidelines, difficulty accessing relevant electronic medical record data at appointments, lack of electronic tools tracking risk, lack of organized resources for provider communication and referrals, low self-efficacy in counseling about behavior change, habit or inertia, fragmentation of care, complexity of health needs, limited patient knowledge, and perception that patients are not interested or capable of acting on recommendations.^18-22^ Patient-level barriers include limited health literacy; lack of awareness; mixed and confusing messages in the media; beliefs that health is determined by a higher power; difficulties balancing health, finances and physical and mental health conditions; and caretaking responsibilities.^4,22,23^ These barriers point to a need for multi-level evidence-based strategies to facilitate CV risk reduction.

For Veterans, CV risk prevention and management have been comparable or even better than among the civilian population,^24-28^ yet gender disparities in CV risk factor control have been identified among Veterans for lipids, blood pressure, and intermediate diabetes outcomes, with women Veterans at elevated risk.^7,8,29-36^ Between 2000–2015, women Veterans experienced rising rates of hypertension (24 to 27%), hyperlipidemia (15 to 25%), diabetes (8 to 11%), and overweight/obesity (10 to 20%), as well as rising rates of depression (27 to 41%) and post-traumatic stress disorder (6 to 18%),^37,38^ which have been identified as risk factors for CV events among women Veterans.^39-42^ As women Veterans are the fastest growing population of Veteran Health Administration (VHA) users,^37^ the prevalence of both traditional CV risk factors and mental health burden makes addressing CV risk a critical VA priority.

To address this need, we worked in collaboration with the VA Office of Women’s Health and VA Greater Los Angeles leadership to identify barriers and facilitators to CV risk identification and reduction among VA providers and their women patients beginning in fiscal year 2013. We then iteratively co-created clinical tools to improve screening of CV risk factors and promote goal-setting for lifestyle changes. This work resulted in the development of a “CV Toolkit” to be used in primary care with women Veterans.^22^ Over time, we have enhanced the CV Toolkit with *implementation strategies*, i.e., “approaches or techniques used to enhance the adoption, implementation, sustainment, and scale-up (or spread) of an innovation,”^43^ specifically, Replicating Effective Programs^43^ enhanced with complexity theory and multilevel stakeholder engagement.

The Enhancing Mental and Physical Health of Women through Engagement and Retention (EMPOWER) Quality Enhancement Research Initiative (QUERI: QUE 15–272) began in 2015 with the goal of implementing innovative care models in VA women’s health to improve women Veterans’ engagement and retention in evidence-based care for three high priority health conditions, i.e., prediabetes, cardiovascular risk, and mental health (depression, anxiety).^44^ The Facilitating Cardiovascular Risk Screening and Risk Reduction in Women Veterans (CV Toolkit; Clinical Trials.gov NCT02991534) project was a component of the EMPOWER QUERI focused on CV risk factors. The goals of the project were to 1) implement and evaluate the CV Toolkit designed to increase CV risk identification and documentation, 2) enhance patient-provider communication about CV risk, and 3) increase women Veterans’ engagement in relevant services and refer them to key VA programs for lifestyle changes. The objective here was to evaluate the effect of CV Toolkit implementation on participation in behavior change programs that target CV risk reduction among women Veterans across five diverse VA sites.

### Description of the CV Toolkit

**Figure 1:**
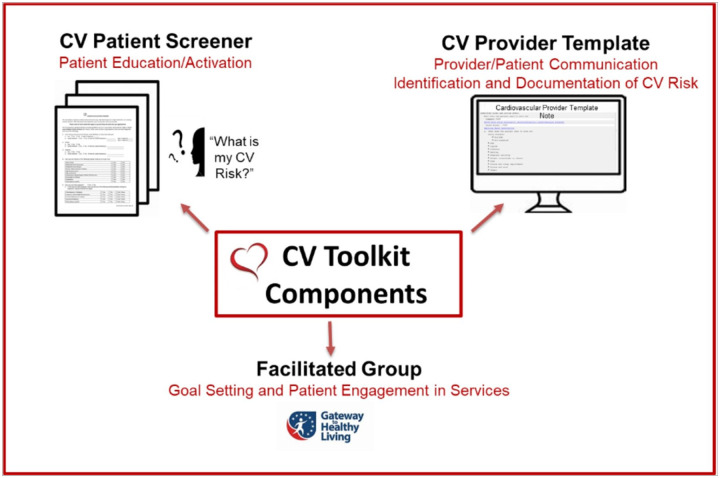
Cardiovascular (CV) Toolkit Components^22^

The CV Toolkit includes three key components (see Fig. 1).^22^ The first component is a patient-facing, one-page CV screener to be given to women at check-in for their primary care appointment. It was originally designed to be given to the patient by the front office staff (i.e., the clerk/scheduler) or the nurse. The CV screener includes a series of questions covering personal health history, family history of CV disease, pregnancy/gestational history, smoking status and physical activity history to encourage a woman to think about her CV risk. The final question asks what she would like to address with her provider at the appointment. The goal of the screener is to help make CV risk discussion a priority for women before they enter the exam room. The second component is a provider CV template that mirrors the patient screener to facilitate CV risk discussion as well as allow for providers to document directly into the electronic health record. The template provides a summary of data available in the electronic health record (e.g., last three entries for weight, blood pressure and cholesterol lab results), in addition to open fields to allow providers to enter any pertinent information from the discussion. The template also includes an action plan section for providers to directly refer patients to relevant programs at their site as well as document recommendations for specific community programs (e.g., YMCA). Risks and details in the template are accessible to other providers to review (e.g., those providers to whom the patient has been referred) within a stand-alone note or embedded into a primary care note. The third component is a gender-tailored group for CV goal setting. The VA National Center for Health Promotion and Disease Prevention’s *Gateway to Healthy Living (Gateway)* is a manualized single-session in-person facilitated group with two follow-up telephone calls. *Gateway* is focused on engaging all Veterans in health behavior change and SMART (specific, measurable, action-oriented, reliable, time-based) goal-setting with discussion of key resources for lifestyle change to prevent chronic disease or optimize health.^45-47^ The CV Toolkit includes a gender-tailored version of *Gateway* that focuses on women-specific CV risk and disease and encourages women to identify their own personal risks. This facilitated group offers time for detailed discussion and SMART goal-setting for health behavior change outside of the time-limited primary care visit, while the subsequent phone visits provide accountability and follow-up. All facilitators of the group were trained in prevention, CV risk, and SMART goal-setting by the VA national office, and facilitators included nurses, peer or health educators, coaches, health psychologists and providers. Follow-up calls were done by the same facilitator that led the in-person group.

### Implementation strategy: the Replicating Effective Programs (REP) framework

**Figure 2:**
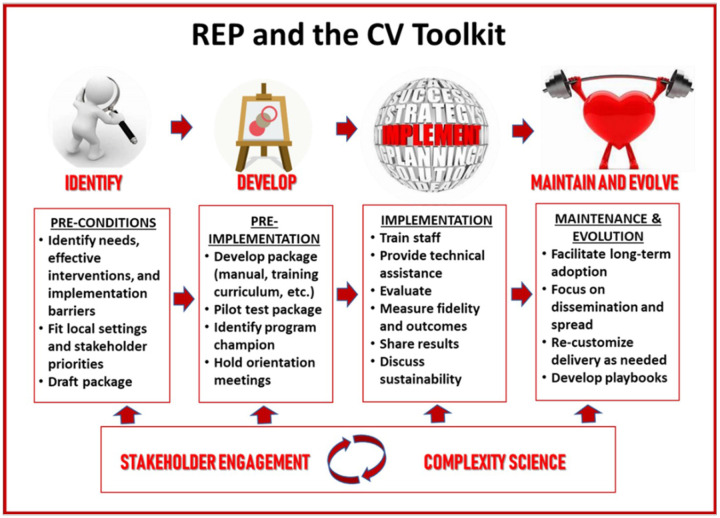
Replicating Effective Practices Framework Phases and the CV Toolkit

The implementation of the CV Toolkit was guided by the Replicating Effective Programs (REP) framework, enhanced with complexity theory and multilevel stakeholder engagement.^44^ An evidence-based, multifaceted implementation strategy,^48^ REP lays out several discrete implementation strategies that correspond to four phases of implementation: pre-conditions, pre-implementation, implementation, and maintenance and evolution.^43^ Complexity theory builds on the insight that outcomes in complex adaptive systems are frequently nonlinear and unpredictable.^44^ In consequence, it is recommended that teams embrace the complexity of multi-level systems, work directly to engage and empower participants across the system, and act both scientifically and pragmatically to develop tailored-to-context solutions as challenges arise.^49^ Every REP phase was therefore informed by multilevel stakeholders, i.e., women Veterans, providers, administrators, and operations partners as well as researchers. Formative *pre-conditions* work built off the prior CV Toolkit development, which indicated that evidence-based strategies need to be combined, tailored, and implemented at the local level to facilitate patient activation and provider-patient discussion while providing accountability and support to women to promote CV risk reduction.^22^ The *pre-implementation* phase included working with our operations partners at the National Center for Health Promotion and Disease Prevention to tailor *Gateway* for women Veterans and CV risk. We conducted in-person site visits to engage with key local partners (ranging from Primary Care or Women’s Health leaders to frontline providers) and consider local adaptations. This phase included identifying champions and tailoring the toolkit to facilitate incorporation into existing clinic workflow, as well as working with sites to determine who would deliver each component of the toolkit. During the *implementation phase*, the toolkit was rolled out at five sites with REP-guided implementation strategies including training and ongoing monthly one-hour technical assistance calls with feedback by the research team. During the *maintenance and evolution phase*, we incorporated feedback from sites and disseminated results to site- and national-level partners. See Fig. 2 for illustration of the REP framework, enhanced with complexity theory and multilevel stakeholder engagement phases for the CV Toolkit.

## METHODS

### Non-randomized Stepped Wedge Design for the Implementation Trial

The stepped wedge design has been a hallmark of implementation studies for more than 20 years.^50^ The design has the advantage, similar to the cross-over design in clinical trials, of allowing for the exposure to both a control and experimental intervention. The control or comparator is simply the period where the study unit is not being exposed to the experimental intercession.

The CV Toolkit evaluation followed a non-randomized stepped wedge design, which relied on a sequential, rather than a random roll-out of the intervention at sites over time.^51^ In stepped wedge designs, randomization of the units to the start of the intervention steps is often considered the gold standard in terms of reduced bias and improved efficiency.^52^ However, randomization of study units is often not feasible for implementation studies, particularly in healthcare service settings.^53^ To evaluate the impact of the CV Toolkit, we used modified steps (rather than randomized steps) given their suitability for evaluating implementation trials because the modified design acknowledges that sites are heterogeneous, face multiple constraints, and are not necessarily ready to adopt interventions at times specified by a randomized design.

The non-randomized stepped wedge capitalizes on the use of data collection at all sites over the entire study period both before and after the start of active implementation. All sites began the observation period in usual care (control state/ “off”), and each site moved into active implementation at some point across the study period (implementation state/“on”). In the statistical evaluation, each site serves as a comparator for both itself and for the implementation states of the other sites at each time point: sites that have not yet started active implementation serve as their own control as wells as serving as controls for sites already in active implementation. The strength of this design is that the modeling of both comparisons (control to intervention for each site as well as between sites) at each time point helps mitigate the concern regarding any site selection bias even though randomization of starting point was not feasible. Also, by incorporating the actual timing of the implementation into the statistical assessment of the intervention effectiveness, the design accounts for potential threats to validity due to historical trends that may occur outside of the intervention. It also accounts for contextual site characteristics that may affect site implementation and performance.

### Implementation Settings

The CV Toolkit was implemented at five VA primary care sites. Sites were eligible if they had at least one women’s health patient-aligned care team (PACT) teamlet (comprising a primary care provider, registered nurse, licensed vocational or practical nurse, and clerk/scheduler), at least one other clinical staff member (e.g., dietitian, pharmacist or mental health provider, etc.), and administrative staff (Women’s Health Medical Director, Women Veterans Program Manager, and Chief of Primary Care or Primary Care Physician Leader). Sites were selected and recruited through the VA Women’s Health Practice Based Research Network.^54^ The five sites were geographically diverse: two in the West, two in the South, and one in the East. Three sites delivered care to women Veterans within comprehensive women’s health clinics in separate, gender-specific space (“Model 3 clinics”), while two sites had mixed gender general primary care clinics with designated women’s health providers (“Model 1 clinics”). The patient panels at the sites ranged from 470–2205 women. ^55^

**Figure 3:**
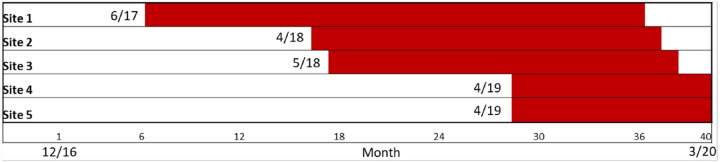
Non-Randomized Stepped Wedge Implementation Schedule

The first site launched in June 2017 and the final site ended in March 2020 (see Fig. 3 for illustration of the implementation schedule). Findings of a mixed-method evaluation assessing uptake of the CV Toolkit intervention across sites have been previously published;^56^ all sites achieved uptake of the intervention during the period of active implementation. Implementation duration varied across sites from 11–29 months. Two sites ended early in March 2020 due to the cessation of in-person primary care services during the COVID-19 pandemic. The project was approved by the VA Central Institutional Review Board.

### Evaluation Sample

All women with at least one primary care visit at one of five primary care sites from December 1, 2016 to March 15, 2020 were eligible to receive the CV Toolkit, allowing for a pre-implementation evaluation of six months prior to the first site beginning implementation (June 2017), and concluding with the service pause for COVID-19. All women VA users within these parameters were eligible, regardless of their Veteran status (e.g., included non-Veteran women patients receiving certain healthcare services who are dependents of disabled Veterans), as all women were potentially at CV risk and could benefit from the toolkit components.

### Measures

The primary goal of CV Toolkit implementation was to increase patient engagement in VA health promotion programs that target the reduction of CV risk. Therefore, we evaluated two specific outcome measures for program engagement, defined as participation in the following VA programs and services: (1) MOVE!, VA’s long-standing health behavior program focused on weight management, and (2) health promotion and disease prevention (HPDP) and/or complementary integrative health (CIH) approaches.^57,58^ Program access for patients varied by site, with some sites requiring consults by providers and other sites relying on patient self-referrals. Therefore, we created a systematic approach incorporating stop codes, consult and note title searches by key words, and individual note review. Primary outcomes were measured at the patient level at each site for each month within the evaluation period (1 = participation during the month versus 0 = no participation during the month). All data were collected from the VA Corporate Data Warehouse.

To identify MOVE! attendance, we used VA service line/stop codes (372 – weight management counseling for individual and 373 – weight management counseling for group), linked consult orders to attendance notes, and searched for additional notes with the key word “move” to capture self-referrals (exclusions for move/movement disorders and bariatric surgery consultations). We corresponded with sites to ensure we credited appropriate groups (e.g., included MOVE Intro, MOVE Group Weekly, etc.; excluded MOVE MD Individual, MOVE Nutrition Individual, etc.). To account for the multi-session format of the program, a patient was eligible for a “new” MOVE! program 35 days from date of her last prior MOVE! visit (based on the distribution curve for attendance where greater than 90% of all attendance happened in less than 35 days). Participation was coded as any new participation in the month (1 = new MOVE! attendance and 0 = no new MOVE! participation).

The second outcome measure captured participation in other VA health promotion programs including HPDP (such as Gateway facilitated group, healthy eating, exercise, sleep, personal development, practice positive thinking, etc.) and/or CIH approaches (such as yoga, Tai Chi, mindfulness meditation). We conducted a comprehensive chart review to identify HPDP and CIH from December 1, 2016, through August 31, 2022. We extracted notes from patients’ electronic health record, which captured attendance to VA programs and services. We reviewed these notes and expanded searching key terms for HPDP and CIH attendance in the note titles that were specific to each study site and CV Toolkit template. These key terms included: integrative health, integrative medicine, gateway, tai chi, yoga, fitness, health promotion, mind over body, healing, healthy, and breathing. After the expanded search, we reviewed the notes and checked the consistency of capturing HPDP and CIH attendance. Participation was coded as yes = 1 for each month with participation in at least one program/service (0 = no participation in any program/service in the month). For the analysis, outcome data was measured for all women from December 1, 2016 through August 31, 2022 to include six-month pre-implementation and six-month post-implementation periods for all sites.

Patient characteristics including age, race (White, Black, American Indian, Asian, Native Hawaiian, and Unknown/Missing), ethnicity (Hispanic/Latina), and military disability service connection were obtained at the start of the observation period. Medical diagnoses related to CV risk documented in the electronic medical record five years prior to baseline included hypertension, hyperlipidemia, and diabetes; we created an indicator of having at least one of those CV risk factor conditions. We also included a diagnosis of overweight/obesity. For mental health conditions, we included documented depression (e.g., major depressive disorder or depression, unspecified) or post-traumatic stress disorder diagnoses in the past two years. Site-level characteristics included the clinic type (i.e., Model 3 versus Model 1 care) and size of site’s panel of women Veterans.

### Analytic model

Our generalized mixed model includes three levels in the hierarchical model: (1) patient, (2) time of intervention (when the site began active implementation) and (3) site. We used Hierarchical Linear Modeling to account for measures at varying hierarchical levels (i.e., patient, time, site),^59^, and we utilized the unit-specific model because it provides predictions for the individual rather than the average individual. One of the main effects in the model was time (Month 1 -Month 45), which is the time trend during which the sites were observed. The second main effect was treatment status (control or active implementation), which indicates if the site was actively implementing or in a control mode. However, the key variable of interest is the time-by treatment interaction, which examines the difference in the time trends for sites in control and then active implementation.^51^ Results are evaluated in terms of direction of the effect of the CV Toolkit being “on” (active) as well as the significance. We used robust standard errors to allow for fewer restrictions on the cluster variability assumption. Based on the skewed distribution in the participation outcomes by age, all models were estimated stratified by age: women younger than 65 years old and women aged 65 and older. Models were estimated using HLM software (Hierarchical linear models).^60^

The power analysis was based on a 3-level hierarchical linear model where patients (level 1) are clustered within time steps (level 2), clustered within sites (level 3). There were nine items considered: α: 0.05; target power (1- β): 0.80 probability of outcome for intervention: 60%; probability of outcome for control: 50%; plausibility for retention for patients who do NOT receive the intervention: 5%-85%; Effect Size Variability: 1%; number of sites: 4; number of time steps per site: 17; and number of patients per time step: 65. These considerations resulted in a sample size of 6009. Note that we exceeded the number of sites (5 instead of required 4) and the number of time steps (44 instead of 17), which makes this calculation conservative.

## RESULTS

A total of 6009 women had at least one primary care visit over the study period. The average age of women was 45 years and 49% of women were white, 32% Black, and 17% Hispanic/Latina. The younger cohort (less than 65 years of age) had higher percentages of Black and Hispanic women compared to the older cohort. Eleven percent of women had diabetes, 30% high cholesterol, 26% hypertension, 41% of the women had at least one of those CV risk factors, and the older cohort had significantly higher rates for all conditions. Twenty-nine percent of women were diagnosed as overweight/obesity. For mental health, 35% had a depression diagnosis, 30% had a post-traumatic stress disorder diagnosis, and both mental health diagnoses were more predominant among the younger women. Seventy-five percent of the women had a service-connected disability (78% of the younger cohort compared to 49% of the older cohort). Most women (96%) seen at the sites were Veterans. Eighty-two percent of the total sample of women were seen in a Model 3 comprehensive women’s health clinic, with this finding higher among the younger cohort of women (82%) compared to the older cohort (76%) (Table 1).

Results from the non-randomized stepped wedge model for each outcome stratified by age are shown in Table 2. For MOVE! participation for women 65 years and older, the interaction effect was significant, indicating that in sites with the CV Toolkit active, women 65 years and older had greater odds (OR = 1.09; CI:1.030,1.152) of participating in the MOVE! program than in sites without the CV Toolkit active (Table 2). For women younger than age 65, the effect was not significant (OR = 1.00; CI:0.976,1.022). In contrast, for HPDP and/or CIH service use, women less than 65 years old who were seen at sites with the CV Toolkit active had greater odds of participation (OR = 1.01; CI = 1.002–1.022) in those programs compared to women in inactive sites.^51^ We conducted a series of sensitivity analyses by testing site-level indicators correlated with variations in uptake (e.g., site size, care delivery model, number of providers using CV Toolkit, and implementation duration). The overall results did not change.

## DISCUSSION

The gender-tailored CV Toolkit intervention was implemented in five diverse clinical sites. Active implementation of the CV Toolkit had a significant effect on participation in behavior change programs. While improvements in participation were found for both age groups, significant improvements varied by program type. Results suggest that program variety may be key to supporting CV targeted behavior change for women across the life span.

A recent scoping review found that relatively few studies in implementation science directly examine client-level outcomes.^61^ Our study, therefore, stands out with its positive, client-level impacts on participation in behavior change programs.^61^ Despite significant variation in clinics’ geographic location, panel size, and models of women’s health care, implementation with enhanced REP achieved adoption of the CV Toolkit at all five participating sites, with significant association between the active implementation of the CV Toolkit and increased lifestyle program participation for women. Women Veterans who were age 65 and older had greater odds of participating in the MOVE! program in the sites when the CV Toolkit was active compared to women at sites when the CV Toolkit was not active, while younger women had greater odds of participating in HPDP/CIH approaches that often include physical activity and stress reduction. Our study demonstrates that REP-guided implementation can achieve program uptake, service delivery improvements, and patient-level outcomes in real-world settings.

Our findings of differential program participation by age underscore the shifting needs and preferences of women Veterans across the life course,^62^ and may help to illustrate how VA and comparable large-scale healthcare systems can benefit from a learning health systems approach.^63^ Implementation and cohort studies with women Veterans over the past decade have demonstrated that VA can adapt, expand, and respond to new challenges, including growing cohorts of aging Veterans.^64-66^ This knowledge is important for ensuring a more responsive health system.^67^ Future research is needed to understand whether other patient characteristics influence choice of health behavior change programs and whether and how these choices or preferences change through the life course. Additionally, understanding the leading health indicators at different life stages is important for prioritizing and optimizing care at different age-specific or condition-specific intervals and allows us to evaluate when and where to insert interventions into frontline or specialty care.^68,69^

During the implementation, we learned more about women’s preferences and struggles with attending in-person programs: all sites reported difficulty with *Gateway* in-person attendance. Women Veterans consistently expressed a desire to attend the gender-tailored group for goal-setting, but many did not show up for the single session. Providers expressed that low group attendance was a common experience at each VA site, and one site requested a telehealth alternative.^70^ Subsequently, we worked with the site and our operations partner to create an adapted telephone version of the goal-setting group. Interest and satisfaction ratings were high among the participants in the pilot program (n = 24). Hence, difficulties with attendance led to an innovation of a telephone-based goal-setting session.

Our findings should be interpreted with several limitations. First, the implementation sites care for a diverse and large number of women Veterans; however, the analytic sample for the model was limited to five sites. The non-randomized stepped wedge accommodates for time trend and history, but implementation start timing for each site was not randomized, which may have introduced bias not accounted for in the model. Also, the power is restricted to the number of sites, which meant we were limited in the number of covariates we could examine in the final models. An additional limitation was the early termination of implementation at two sites due to COVID-19. One site limited care to urgent needs and one site closed during the pandemic. In all sites, primary care and health promotion programs were modified, and health promotion and behavior change programs were often delayed with the transition to virtual options. Therefore, post-implementation data reflect these unanticipated and unprecedented changes. Another limitation is that for programs requiring a consult order, scheduling by phone could have led to errors or losses to follow-up scheduling which would likely bias our results to the null. Finally, the analysis does not account for variations in uptake of the individual CV Toolkit components. Specifically, the patient-facing screener was administered by the site and not collected for research. Despite these limitations, we still found significant impact of CV Toolkit implementation on engagement in relevant services.

This work represents a learning health system in action.^71^ First, national operations partners (VA Office of Women’s Health) identified a clear need: to address high CV risk among women Veterans. Then, collaboration between researchers and operations partners led to the identification of the organizational-, provider- and patient-level barriers to and facilitators of CV risk identification, documentation, discussion, and reduction, which in turn led to the development of focused clinical tools.^22^ The VA Quality Enhancement Research Initiative^72^ then funded the implementation of the tools into practice, where we saw an improvement in engagement in focused health behavior change programs.

Through implementing the CV Toolkit as well as the other components of EMPOWER focused on diabetes prevention and collaborative care for women’s mental health, we learned that women prefer to engage in interventions that are convenient and sensitive to their life circumstances, especially since a substantial proportion of women are isolated from care due to competing responsibilities, rurality and/or urban isolation, as well as disabilities and histories of trauma or gender-bias.^22,66,73-75^ We also found specifically with our work on *Gateway* that many women favor virtual delivery when presented with in-person and virtual modality options, and that gender tailoring is a critical factor for engagement.^66^

## CONCLUSIONS

Refining, implementing, and evaluating the innovative CV Toolkit to facilitate appropriate screening, discussion, and documentation of CV risk was an important step in improving patient-provider conversations about CV risk factors and led to actionable steps to manage risk factors. CV Toolkit use at the clinic level helped achieve the EMPOWER QUERI goal of improving engagement and retention of women Veterans in patient-centered, proactive, personalized care to address CV risk. Within the context of women’s primary care, the packaged toolkit components encouraged Veterans to partner with their providers and become more active participants by promoting and documenting communication interactions, building in local choices for lifestyle change, and prompting an action step for health goals.

## Figures and Tables

**Table 1:** Characteristics of women seen in primary care at the five implementation sites

		Women younger	Women 65	
	Total Sample	than age 65	years & older	
Characteristic	(N = 6009)	(N = 5496)	(N = 540)	P-value
Mean age (SD)	45.6 (14.7)	43.0 (12.4)	72.6 (7.5)	< 0.0001
Race				< 0.0001
White	2954 (49.2%)	2630 (48.1%)	324 (60.0%)	
Black	1905 (31.7%)	1768 (32.3%)	137 (25.4%)	
Asian, NH, OPI, AI or, AN*	376 (6.3%)	358 (6.6%)	18 (3.3%)	
Unknown	774 (12.9%)	713 (13.0%)	61 (11.3%)	
Ethnicity				< 0.0001
Hispanic or Latina	1011 (16.8%)	973(17.8%)	38 (7.0%)	
Not Hispanic or Latina	4742 (78.9%)	4265 (78.0%)	477 (88.3%)	
Unknown	256 (4.3%)	231 (4.2%)	25 (4.6%)	
Diagnosis in last 5 years				
Diabetes	677 (11.3%)	521 (9.5%)	156 (28.9%)	< 0.0001
High cholesterol	1786 (29.7%)	1415 (25.9%)	371 (68.7%)	< 0.0001
Hypertension	1585 (26.4%)	1207 (22.1%)	378 (70.0%)	< 0.0001
Has at least 1 of 3 CV risk factors**	2432 (40.5%)	1968 (36.0%)	464 (85.9%)	< 0.0001
Overweight/Obesity	1769 (29.4%)	1595 (29.2%)	174 (32.2%)	0.137
Mental Health diagnosis in last 2 years				
Depression	2090 (34.8%)	1940 (35.5%)	150 (27.8%)	< 0.0001
PTSD	1815 (30.2%)	1724 (31.5%)	91 (16.9%)	< 0.0001
Service Connected	4515 (75.1%)	4250 (77.7%)	265 (49.1%)	< 0.0001
Veteran	5757 (95.8%)	5221 (95.5%)	536 (99.3%)	< 0.0001
% Seen in WH clinic (vs PC)***	4909 (81.7%)	4499 (82.3%)	410 (75.9%)	< 0.0001

* Asian, Native Hawaiian, Other Pacific Islander, American Indian, or Alaska Native

** At least one of the following CV risk factors: hypertension, high cholesterol, and diabetes

*** Percent of women seen in a comprehensive women's health clinic (Model 3) versus general primary care (Model 1)

**Table 2:** Intervention effectiveness stratified by age^1^

Attendance at MOVEI Exercise Program for women age 65 and older			
	Odds Ratio	Confidence Interval	P value
Intercept	0.01	(0.005,0.009)	<0.001
Time	0.87	(0.838,0.908)	<0.001
CV Toolkit Intervention	1.74	(0.707,4.280)	0.228
Interaction	**1.09**	**(1.030,1.152)**	**0.003**
Attendance at MOVEI Exercise Program for women less than 65 years old			
	Odds Ratio	Confidence Interval	P value
Intercept	0.00	(0.002,0.007)	<0.001
Time	0.99	(0.974,1.003)	0.114
CV Toolkit Intervention	1.28	(0.820,1.985)	0.28
Interaction	1.00	(0.976,1.022)	0.91
Attendance at HPDP programs and/or CIH service use for women age 65 and older			
	Odds Ratio	Confidence Interval	P value
Intercept	0.00	(0.001,0.016)	<0.001
Time	0.96	(0.892,1.040)	0.34
CV Toolkit Intervention	0.39	(0.066,2.297)	0.298
Interaction	1.06	(0.959,1.165)	0.265
Attendance at HPDP programs and/or CIH service use for women less than 65 years old			
	Odds Ratio	Confidence Interval	P value
Intercept	0.01	(0.003,0.027)	<0.001
Time	1.00	(0.993,1.010)	0.715
CV Toolkit Intervention	0.79	(0.653,0.953)	0.014
Interaction	**1.01**	**(1.002,1.022)**	**0.015**

1 Fixed effects (Unit-specific model) with robust standard errors

## Abbreviations

CV: Cardiovascular
CIH: complementary integrative health
EMPOWER: Enhancing Mental and Physical Health of Women through Engagement and Retention
HPDP: health promotion and disease prevention
HLM: Hierarchical linear models
PACT: patient-aligned care team
QUERI: Quality Enhancement Research Initiative
REP: Replicating Effective Programs
SMART goals: specific, measurable, action-oriented, reliable, time-based
VA: Veteran Health Administration
